# Nupr1 Modulates Methamphetamine-Induced Dopaminergic Neuronal Apoptosis and Autophagy through CHOP-Trib3-Mediated Endoplasmic Reticulum Stress Signaling Pathway

**DOI:** 10.3389/fnmol.2017.00203

**Published:** 2017-06-26

**Authors:** Xiang Xu, Enping Huang, Yunchun Tai, Xu Zhao, Xuebing Chen, Chuanxiang Chen, Rui Chen, Chao Liu, Zhoumeng Lin, Huijun Wang, Wei-Bing Xie

**Affiliations:** ^1^School of Forensic Medicine, Southern Medical UniversityGuangzhou, China; ^2^School of Forensic Medicine, Wannan Medical CollegeWuhu, China; ^3^Department of Forensic Medicine, Guangdong Medical UniversityDongguan, China; ^4^Guangzhou Forensic Science InstituteGuangzhou, China; ^5^Institute of Computational Comparative Medicine and Department of Anatomy and Physiology, College of Veterinary Medicine, Kansas State UniversityManhattan, KS, United States

**Keywords:** methamphetamine, Nupr1 (Nuclear protein 1/com1/p8), endoplasmic reticulum stress, neurotoxicity, apoptosis, autophagy

## Abstract

Methamphetamine (METH) is an illegal and widely abused psychoactive stimulant. METH exposure causes detrimental effects on multiple organ systems, primarily the nervous system, especially dopaminergic pathways, in both laboratory animals and humans. In this study, we hypothesized that Nuclear protein 1 (Nupr1/com1/p8) is involved in METH-induced neuronal apoptosis and autophagy through endoplasmic reticulum (ER) stress signaling pathway. To test this hypothesis, we measured the expression levels of Nupr1, ER stress protein markers CHOP and Trib3, apoptosis-related protein markers cleaved-caspase3 and PARP, as well as autophagy-related protein markers LC3 and Beclin-1 in brain tissues of adult male Sprague-Dawley (SD) rats, rat primary cultured neurons and the rat adrenal pheochromocytoma cells (PC12 cells) after METH exposure. We also determined the effects of METH exposure on the expression of these proteins after silencing Nupr1, CHOP, or Trib3 expression with synthetic small hairpin RNA (shRNA) or siRNA *in vitro*, and after silencing Nupr1 in the striatum of rats by injecting lentivirus containing shRNA sequence targeting Nupr1 gene to rat striatum. The results showed that METH exposure increased Nupr1 expression that was accompanied with increased expression of ER stress protein markers CHOP and Trib3, and also led to apoptosis and autophagy in rat primary neurons and in PC12 cells after 24 h exposure (3.0 mM), and in the prefrontal cortex and striatum of rats after repeated intraperitoneal injections (15 mg/kg × 8 injections at 12 h intervals). Silencing of Nupr1 expression partly reduced METH-induced apoptosis and autophagy *in vitro* and *in vivo*. These results suggest that Nupr1 plays an essential role in METH-caused neuronal apoptosis and autophagy at relatively higher doses and may be a potential therapeutic target in high-dose METH-induced neurotoxicity.

## Introduction

Methamphetamine (METH) is an illicit and one of the most widely used addictive central nervous system stimulants. In both laboratory animals and humans, METH exposure causes extensive pathological alterations to multiple organ systems, such as the brain, heart and lungs, with the brain being the primary target (Carvalho et al., 2012; Vearrier et al., 2012; Halpin et al., 2014). Neuroimaging analyses have shown that METH users exhibit pathological changes, including smaller gray matter volumes and white matter hypertrophy in several brain regions, such as prefrontal cortex, striatum, hippocampus and midbrain (Thompson et al., 2004; Chang et al., 2007; Berman et al., 2008; Schwartz and Dell, 2010; Nakama et al., 2011; Grant et al., 2012). Epidemiological studies suggest that METH use is a significant risk factor that increases the susceptibility to neurodegenerative diseases, such as Parkinson’s disease (Garwood et al., 2006; Callaghan et al., 2012). However, the molecular mechanisms of METH’s role in the etiology of PD-like pathology remain to be elucidated.

In the brain, METH mainly targets the dopaminergic system, and other types of brain cells can also be affected. For example, it has been shown that METH exposure causes neuronal death in several brain areas, such as the striatum, indusium griseum, medial habenular nucleus and amygdale (Bowyer and Ali, 2006; Kitamura et al., 2007). In addition to these results observed *in vivo*, other studies, including ours, have demonstrated that METH exposure can cause apoptosis and autophagy in dopaminergic neurons (Castino et al., 2008; Kongsuphol et al., 2009; Huang et al., 2015; Chen C. et al., 2016; Roohbakhsh et al., 2016; Li et al., 2017). However, the mechanisms underlying METH-induced apoptosis and autophagy in neuronal cells have not yet been elucidated.

The endoplasmic reticulum (ER) is a vital organelle in eukaryotic cells (Díaz-Villanueva et al., 2015). A number of intracellular and extracellular factors, such as exposure to toxicants can disturb the homeostasis of ER, leading to ER stress (Schröder, 2008). Growing evidence suggests that ER stress is involved in the apoptosis and autophagy caused by METH treatment (Krasnova and Cadet, 2009; Abekawa et al., 2011; Takeichi et al., 2012; Cai et al., 2016; Li et al., 2017). Our recent results indicate that ER stress mediated by Nuclear protein (Nupr1) plays a crucial role in METH-induced vascular endothelial cell apoptosis (Cai et al., 2016). Nupr1 is also involved in Stx2-related autophagic cell death via the ER stress pathway in intestinal epithelial cells (Tang et al., 2015). Another recent study showed that Nupr1 can regulate autophagy via mTOR signaling pathway in cancer cells (Jia et al., 2016). Nupr1 is one of the proteins related to the high mobility group of transcriptional regulators. It is a critical player in the cellular stress and is involved in metastasis. Nupr1 was first identified as a gene induced in pancreatitis, but has since then been found over-expressed in several types of cancer and pathological conditions. Despite its small size and relatively simple structure, Nupr1 functions in several genetic and biochemical signaling pathways. Nupr1 expression is low in physiological condition, but can be induced by hypoxia, oxidative stress, DNA damage and other stimuli and plays an important role in these biological processes (Ree et al., 1999, 2000; Carracedo et al., 2006; Chowdhury et al., 2009). However, the role of Nupr1 in METH-induced neurotoxicity has not been reported.

CHOP, as an ER stress marker protein (Cai et al., 2016), was first identified to be a member of the CCAAT/enhancer binding proteins (C/EBPs) that serves as a dominant negative inhibitor of C/EBPs. CHOP is also known as growth arrest- and DNA damage-inducible gene 153 (GADD153), DNA–damage-inducible transcript 3 (DDIT3) and C/EBPζ. CHOP is ubiquitously expressed at very low levels, but it is robustly expressed by perturbations that induce stress in a wide variety of cell types (Ron and Habener, 1992). CHOP plays an important role in ER stress-induced apoptosis (Wang et al., 1996; Zinszner et al., 1998; Maytin et al., 2001; McCullough et al., 2001; Oyadomari et al., 2001; Gotoh et al., 2002), and accumulated evidence also shows that CHOP-mediated apoptosis may also be involved in the development of Parkinson’s disease (Iadecola et al., 1997; Katayama et al., 1999; Sato et al., 2000). Recently, it was reported that upregulated expression of CHOP and induction of apoptosis in response to ER stress can be directly induced by Nupr1 in PANC-1 human pancreatic carcinoma cells (Khalyfa et al., 2007; Palam et al., 2015). Whether Nupr1/CHOP pathway is involved in METH-induced apoptosis via ER stress in dopaminergic neuronal cells remains elusive.

Trib3 (tribbles homolog 3), as a novel ER stress-inducible gene, is involved in autophagic cell death via induction of ER stress, activation of the unfolded protein response (UPR), and through mTOR signaling pathway (Ohoka et al., 2005; Rubiolo et al., 2014). Previous studies showed that upregulating the expression of Trib3 can inhibit the interaction of AKT (protein kinase B) with upstream kinases, leading to inhibition of AKT/mTORC1 axis and autophagy-mediated cell death (Salazar et al., 2015; Erazo et al., 2016). ER stress can promote autophagy via Trib3-dependent inhibition of the AKT/mTORC1 axis in cannabinoid-induced human and mouse cancer cell death (Salazar et al., 2009). However, whether and how Nupr1/Trib3 pathway exerts a modulatory function on autophagy via ER stress in dopaminergic neuronal cells by METH exposure remains to be investigated.

The objective of this study was to investigate the mechanisms of METH-induced apoptosis and autophagy in dopaminergic cells, mainly focusing on the role of Nupr1 and ER stress in this process. We hypothesized that Nupr1 may mediate METH-induced apoptosis and autophagy in dopaminergic cells through ER stress pathway and knockdown of Nupr1 expression could partially protect against METH-induced apoptosis and autophagy. In order to test this hypothesis, we measured Nupr1, ER stress, apoptosis and autophagy-related marker protein levels in rat adrenal pheochromocytoma PC12 cells, rat primary cultured neurons, and in brain tissues of rats after METH exposure. We also evaluated the effects on METH-caused changes in the expression of selected protein markers after silencing Nupr1 expression with synthetic small hairpin RNA (shRNA) or siRNA *in vitro*, and by injecting lentivirus containing shRNA sequence targeting Nupr1 gene to rat striatum. Our results demonstrated that METH exposure increased Nupr1 expression and activated ER stress-related proteins CHOP and Trib3 expression both *in vitro* and *in vivo*, as well as reduced phosphorylation of mTOR in PC12 cells. These effects were partially normalized after inhibiting Nupr1. We conclude that Nupr1 plays a crucial role in the regulation METH-induced apoptosis and autophagy partly through ER stress signaling pathway in neuronal cells and Nupr1 promotes neuronal cell autophagy mainly through the inhibition of p-mTOR activity. The Nupr1/CHOP/Trib3 pathway may be a potential therapeutic target of METH-induced neurotoxicity.

## Materials and Methods

### Animal Protocol

Healthy adult male Sprague-Dawley (SD) rats (180–220 g, 6–8 weeks old) were purchased from Laboratory Animal Center of Southern Medical University (Guangzhou, China) and housed singly in tub cages on a temperature-controlled room on a 12 h light-12 h dark cycle with food and water available *ad libitum*. Rats were habituated to the animal room for 1 week before use and divided randomly into three groups (*n* = 3/group): saline control group, subacute exposure group and chronic exposure group. Rats in the control and subacute exposure groups were exposed via intraperitoneal injection (i.p.) to saline vehicle or 15 mg/kg METH (>99% purity; National Institutes for Food and Drug Control, Guangzhou, China) for eight injections at 12 h intervals, respectively. This subacute exposure paradigm is relevant to human short-term exposure based on several previous studies because the measured concentrations of METH in the blood and brain of rats at 1 h after the last injection were in the range of reported blood concentrations (0.6–5 μg/ml [4–30 μM]) in humans (Winek et al., 2001; Qiao et al., 2014; Huang et al., 2015). In the chronic exposure group, the animals received i.p. injections of METH following the dosing schedule listed in Table 1 as reported in earlier studies (Kobeissy et al., 2012; Cai et al., 2016; Li et al., 2017). This chronic exposure paradigm was chosen because it can mimic the behavior of long-term METH use in humans and the level of dopamine transporter was decreased in the cortex and striatum of rats after the chronic METH exposure (Danaceau et al., 2007; Li et al., 2017). Rats were euthanized at 24 h after the last injection, brain samples were removed quickly and the samples (prefrontal cortex and striatum tissues) were dissected on ice and stored at −80°C until use. We selected the prefrontal cortex and striatum because these two brain regions have been shown to be target sites of METH (Hsu et al., 2008; Lin et al., 2013; Long et al., 2017). Frozen tissue sections (5 μm in thickness) were sliced using a freezing microtome (CM1900, Leica, Wetzlar, Germany) for subsequent staining and immunofluorescence labeling. All animal procedures were carried out according to the NIH Guidelines for the Care and Use of Laboratory Animal (8th Edition, U.S. National Research Council, 2011) and were approved in advance by the Institutional Animal Care and Use Committee at the Southern Medical University.

**Table 1:** Dosing schedule of Methamphetamine (METH) in the chronic exposure group (mg/kg).

Injection time	Day 1	Day 2	Day 3	Day 4	Day 5	Day 6	Day 7	Day 8	Day 9	Day 10	Day 11	Day 12	Day 13	Day 14
8:00	1.0	1.0	1.0	1.0	1.5	1.5	2.0	2.0	2.5	3.0	3.5	4.0	4.5	5.0
10:00				1.0	1.5	1.5	2.0	2.0	2.5	3.0	3.5	4.0	4.5	5.0
12:00				1.0	1.5	1.5	2.0	2.0	2.5	3.0	3.5	4.0	4.5	5.0
14:00		1.0	1.0	1.0	1.5	1.5	2.0	2.0	2.5	3.0	3.5	4.0	4.5	5.0

### Cell Culture

The rat adrenal pheochromocytoma PC12 cell line (purchased from Shanghai Cell Bank of Chinese Academy of Sciences) was selected as an *in vitro* model because it can synthesize and store dopamine and is widely used in studying dopaminergic toxicity mechanism (Greene and Tischler, 1976; Anantharam et al., 2002; Huang et al., 2015). PC12 cells were cultured in DMEM medium containing 10% fetal bovine serum (Gibco, Grand Island, NY, USA), and maintained at 37°C in a humidified atmosphere of 5% CO_2_. The cells were passaged every 3–4 days. PC12 cells were initially exposed to a concentration range of METH 0.5–3.5 mM for 24 h to evaluate the dose-dependence of METH’s effects on the expression of Nupr1, CHOP and Trib3. The concentration of 3.0 mM for PC12 cells was selected for subsequent experiments based on the LC25 of METH in this cell type and based on significant alterations of Nupr1, ER stress-, apoptosis- and autophagy-related marker protein levels at this concentration (Huang et al., 2015). Next, we exposed PC12 cells to 3.0 mM METH for 0 h, 2 h, 4 h, 8 h, 16 h and 24 h to evaluate the time-dependence of METH’s effects on the expression of Nupr1, CHOP, and Trib3. These *in vitro* concentrations are in line with earlier studies from our lab (Huang et al., 2015; Chen C. et al., 2016; Li et al., 2017) and other groups (Cadet et al., 1997) to allow for comparisons between different studies.

### Primary Culture of Prefrontal Cortex and Striatal Neurons from Sprague-Dawley (SD) Rats

Primary rat prefrontal cortex and striatal neuronal culture was prepared as previously described (Finkbeiner et al., 1997; Saudou et al., 1998; Dong et al., 2012; Lepsch et al., 2015). In brief, rat fetuses (embryos on embryonic day 16–18) were harvested by cesarean section from anesthetized pregnant SD rats after cleaning the skin with 75% alcohol, and then transferred to a 35 mm Petri dish containing cold CMF-HBSS (calcium- and magnesium-free Hank’s balanced salt solution, 4°C). Fetal brains were isolated under a dissection microscope and transferred to another 35 mm Petri dish containing cold CMF-HBSS (4°C), then the bilateral prefrontal cortex and striatum were dissected out and transferred to a new 35 mm dish also containing cold CMF-HBSS (4°C). The dissected tissues were minced in 1 mm^3^ size pieces using a curved scissor, and then transferred to a 15 mL conical tube. The digestion procedure was performed by adding 3–5 ml 0.25% trypsin-EDTA (Gibco) and the cells were incubated at 37°C in a humidified atmosphere containing 5% CO_2_ for 10 min. After that, 10 ml DMEM/F12 (1:1) medium supplemented with 10% FBS was added to the tube to terminate the digestion, followed by gentle mixing with a glass Pasteur pipette, making sure the tissues pass through the pipette gently. The mixture containing individual cells was collected and transferred into another 50 ml falcon tube, and then centrifuged at 1000 rpm for 5 min. The supernatant was removed and the tissue pellet was resuspended in 10 ml neurobasal medium containing 2% B27, 1% Glutamax-100X and 5 μM glutamate (Gibco). After standing for 1–2 min, the supernatant liquid was withdrawn in order to count the cell number. Cells were plated in six-well plates (1–2 × 10^6^ cells/well), which were precoated with 0.01% poly-L-lysine (Sigma, St. Louis, MO, USA), and incubated with 2 ml neurobasal medium supplemented with 2% B27, 1% Glutamax-100X and 1 μM glutamate. Fifty percent of the medium was changed 3 days after plating and subsequently every 2 days with neurobasal medium supplemented with 2% B27. Neuronal cultures were maintained for up to 12–14 days *in vitro* (DIV12) and then used for Western blot and immunofluorescence analyses.

### Immunofluorescence Labeling

For immunofluorescence staining of frozen tissue sections and primary cultured neurons, all sample incubation solutions were prepared using phosphate-buffered saline (PBS) supplemented with 10% goat serum and 0.1% Triton X-100. The rabbit polyclonal anti-p8 antibody (1:200, Santa Cruz, Dallas, TX, USA) and the fluorescein (FITC)-conjugated goat anti-rabbit IgG antibody (1:50, DingGuo, Dalian, China) were used together with (4′,6′-diamidino-2-phenylindole, DAPI) for nuclear labeling. We used anti-NeuN antibody (1:1000, rabbit, Abcam, Cambridge, UK) as neuronal marker to detect neurons (data not shown). The frozen tissue section samples were incubated with blocking buffer for 30 min at room temperature and then with primary antibody at 4°C overnight. After washing with PBS for three times, the samples were incubated with secondary antibody for 1 h at room temperature. Microphotographs were taken using a fluorescence microscopy (A1+/A1R+, Nikon, Tokyo, Japan). All digital images were processed using the same settings to improve the contrast.

### RT-QPCR

Total RNA was extracted using RNAisoPlus Kit after METH treatment for 24 h. Thereafter, cDNA was reversely transcribed from 1 μg of total RNA following the PrimeScript™ RT reagent Kit and SYBRs Premix ExTaq™ Kit instructions. Primers were designed by GenePharma Co., Ltd. (Suzhou, China). Sequences of Nupr1 primers were as follows: forward, 5′AGCCTGGCCCAATCTTATGT3′ and reverse, 5′GGCCTAGGTCCTGCTTACAA3′. Sequences of CHOP primers were as follows: forward, 5′AAATAACAGCCGGAACCTGA 3′ and reverse, 5′CGTTTCCTGGGGATGAGATA 3′. Sequences of GAPDH primers were as follows: forward, 5′GTGAAGGTCGGAGTCAACG 3′ and reverse, 5′TGAGGTCAATGAAGGGGTC3′.

### Western Blotting

Brain tissues and cells were lysed in RIPA buffer with protease and phosphorylase inhibitors at 4°C for 30 min. Protein concentrations were measured with the BCA-100 Protein Quantitative Analysis kit (Biocolors, Shanghai, China). Protein samples (10 μg) were separated by 6%–12% sodium dodecyl sulfate polyacrylamide gel (SDS-PAGE) and transferred onto polyvinylidenedifluoride (PVDF) membranes (Millipore, Billerica, MA, USA). The membranes were incubated at room temperature for 1 h in blocking buffer (5% nonfat dry milk in TBST, 1× TBS + 0.1% Tween20 buffer), followed by incubation with diluted primary antibodies overnight with gentle shaking at 4°C. Anti-p8 antibody (sc-30184; 1:500 dilution), anti-CHOP antibody (sc-575; 1:500 dilution), anti-TRB3 antibody (sc-365842; 1:500 dilution) were obtained from Santa Cruz Biotechnology, Inc. (Dallas, TX, USA). Anti-PARP antibody (#9532S; 1:1000 dilution), anti-cleaved caspase-3 antibody (#9664S; 1:1000 dilution), anti-Beclin-1 antibody (#3495S; 1:1000 dilution), anti-LC3A/B antibody (#12741S; 1:1000 dilution), anti-Phospho-mTOR antibody (Ser2448; #2971S; 1:1000 dilution), anti-mTOR antibody (#2972S; 1:1000 dilution), anti-Phospho-AKT antibody (Ser473; #4060S; 1:1000 dilution), and anti-β-actin antibody (#4970L; 1:1000 dilution) were purchased from Cell Signaling Technology, Inc. (Boston, MA, USA). Membranes were washed three times with TBST buffer and then incubated with corresponding horse radish peroxidase (HRP)-conjugated secondary antibodies at room temperature (25°C) for 1 h. The membranes were developed with Chemiluminescence ECL^Plus^ Western Blotting detection reagents and the signal of band intensities was quantitated with Gel-Pro analyzer (Media Cybernetics, Inc., Rockville, MD, USA). The level of β-actin was utilized as a reference control. For each protein of interest, we repeated three independent experiments, and selected the representative band shown in this manuscript.

### RNA Interference and Transfection

Two pieces of shRNA, two pieces of siRNA, and two pieces of siRNA (GenePharma Co., Ltd., Suzhou, China) sequences targeting Nupr1, CHOP and Trib3, respectively, were designed as shown below: Nupr1 shRNA #1 (Rat, GCAACCTGTAAACATAGAG), shRNA #2 (Rat, TCCTGGATGAGTATGA CCA); CHOP siRNA #1 (Rat, 5′-GGGAUACCAUGCAACAUAATT-3′), siRNA #2 (Rat, 5′-CUA GAAAUCUGUUGCUAUGTT-3′), as well as Trib3 siRNA #1 (Rat, 5′-GCUCUCAGCUCCUAUAC ACTT-3′) and siRNA #2 (Rat, 5′-GCAACUGUGAGAGGA CGAATT-3′) for PC12 cells. Cells seeded on a 6-well plate were grown to 70%–90% confluence. Lipofectamine 3000 (Invitrogen, Carlsbad, CA, USA) reagent and 100 nmol siRNA were mixed with Opti-MEM medium (Invitrogen, Carlsbad, CA, USA). The mixed solution was vortexed for 2–3 s, and then incubated for 5 min at room temperature prior to adding the mixed solution to cells for another 4–6 h incubation. After that, the siRNA/Lipofectamine 3000 complex medium was replaced with the same volume of regular FBS-supplemented culture medium. After 48 h incubation, the regular medium was changed to non-serum medium prior to treatment with METH.

### TUNEL Staining

After 24 h incubation of PC12 cells (2–5 × 10^5^/dish), cells were pretreated with shRNA #2 targeting Nupr1 or control shRNA (Rat, TCCTGGATGAGTATGACCA), and then exposed to METH (3.0 mM) or vehicle for 24 h. We used the fluorometric TUNEL system (EMD Millipore Corporation, Billerica, MA, USA) to detect DNA fragmentation of apoptotic cells according to the manufacturer’s instructions. Briefly, PC12 cells were fixed in 1% paraformaldehyde in fresh PBS (pH 7.4) at room temperature for 10 min, incubated with fluorescein-conjugated TdT enzyme at 37°C for 1 h in the dark, and then mounted with DAPI for nuclear counter staining. Cross-sections were imaged (20× objective) using a fluorescence microscope (Nikon, Tokyo, Japan). The control incubation buffer was prepared without the TdT enzyme; all other steps were similar. Samples were stained with DAPI to determine the total number of nuclei. Both TUNEL- and DAPI-positive cells were counted. Data are presented as the TUNEL index, which was calculated based on the total number of TUNEL-positive cells.

### shRNA and Stereotaxic Injection

The synthesis of shRNA and stereotaxic injection protocol were based on our recent studies (Huang et al., 2015; Chen R. et al., 2016; Li et al., 2017). In brief, the Nupr1 shRNA #2 sequence (TCCTGGATGAGTATGACCA) was cloned into pGC-LV vector and transfected into HEK293FT cells with pHelper 1.0 and 2.0 vectors. LV-shNupr1 lentivirus was harvested with 10^9^ transducing units per milliliter. LV-GFP was used as the control virus. Healthy adult male SD rats (200–220 g) were divided randomly into four groups (*n* = 3/group): LV-GFP group, LV-shNupr1 group, LV-GFP + METH group, and LV-shNupr1 + METH group. The animals were anesthetized via i.p. injection with 1% pentobarbital before surgical procedures. The anesthetized rats were fixed in a stereotaxic frame (Domitor; Wood Dale, IL, USA), and an incision was made on the skin overlying the skull. A 10 μl Hamilton syringe was used to inject 5 μl of LV-shNupr1 lentivirus or LV-GFP lentivirus at a rate of 0.25 ml/min to the right striatum at the following stereotaxic coordinates: 1.0 mm rostral to bregma, 3.0 mm lateral to the midline (right side), 4.5 mm ventral to the dura, with tooth bar set at zero. The striatum instead of prefrontal cortex was selected to investigate the effect of silencing Nupr1 expression on METH-induced apoptosis and autophagy *in vivo* because METH primarily targets dopaminergic neurons and striatum has been shown to be METH’s major target site, where the densities of dopaminergic synapses are the highest, whereas dopamine terminals in the prefrontal cortex are relatively sparse compared to the striatum (McCann et al., 1998; Ernst et al., 2000; Volkow et al., 2001; Chang et al., 2005, 2007). After injection, the cannula remained *in situ* for an additional 4 min before being withdrawn slowly and gently. After 4 days of recovery, animals were treated with saline vehicle or METH (15 mg/kg × 8 injections, at 12 h intervals) via i.p. injection and euthanized at 24 h after the last injection. Brain samples were quickly removed and the striatum tissues were dissected on ice-cold glass plate, frozen rapidly and then stored at −80°C until use.

### Statistical Analysis

All data are summarized as mean ± standard deviation (SD) with at least three independent replicates. Statistical analysis was conducted by using one-way ANOVA followed by least significant difference (LSD) *post hoc* analysis or independent-samples *t*-test (as appropriate) using the scientific statistic software SPSS version 19.0 (SPSS Inc., Chicago, IL, USA). A *p* value of <0.05 was considered statistically significant.

## Results

### METH Increases Nupr1 and CHOP mRNA and Protein Expression in PC12 Cells

**Figure 1:**
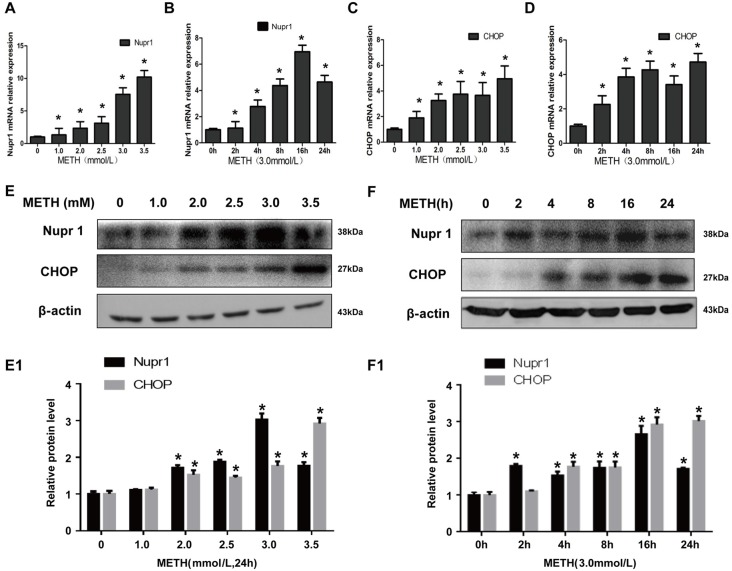
Methamphetamine (METH) increases nuclear protein 1 (Nupr1) and CHOP mRNA and protein expression in PC12 cells. METH exposure up-regulates Nupr1 and CHOP mRNA and protein expression in a concentration- and time-dependent manner in PC12 cells. PC12 cells were exposed to 1.0 mM, 2.0 mM, 2.5 mM, 3.0 mM and 3.5 mM METH for 24 h **(A,C,E)** and 3.0 mM METH for 2 h, 4 h, 8 h, 16 h and 24 h **(B,D,F)**. RT-QPCR **(A–D)** was performed to determine Nupr1 and CHOP mRNA expression; Western blot **(E,F)** and quantitative analyses **(E1,F1)** were performed to determine Nupr1 and CHOP protein expression. β-actin was used as a loading control. Fold induction relative to cells treated with vehicle is shown. *p < 0.05 vs. vehicle-treated cells. Data were analyzed with one-way ANOVA followed by least significant difference (LSD) *post hoc* analyses. Data are expressed as means ± standard deviation (SD; *n* = 3/group).

In order to analyze how METH affects Nupr1 and CHOP expression, PC12 cells were treated with a dose range (1.0–3.5 mM) of METH for 24 h or treated with 3.0 mM METH for 2 h, 4 h, 8 h, 16 h and 24 h. The RT-QPCR and western blot results showed that METH increased Nupr1 and CHOP mRNA levels (Figures 1A–D) and protein expression in a dose-dependent (Figures 1E,E1) and time-dependent (Figures 1F,F1) manner. For example, after 24 h exposure, the Nupr1 mRNA level and protein expression were significantly increased by 4.84-fold and 3.17-fold in the METH-treated (3.0 mM) PC12 cells, respectively (*n* = 3, *p < 0.05). Similarly, CHOP mRNA level and protein expression were significantly increased by 4.97-fold and 1.81-fold in the 24 h METH-treated (3.0 mM) cells, respectively (*n* = 3, *p < 0.05). However, Nupr1 and CHOP protein expression levels were not significantly changed by 1.0 mM METH after 24 h exposure. Therefore, increased Nupr1 and CHOP expression by relatively higher doses of METH may partly contribute to high-dose METH-induced neurotoxicity, otherwise, it appears that Nupr1 and CHOP are not involved in low-dose METH-induced neurotoxicity.

### METH Increases Nupr1 and CHOP Protein Expression in Rat Primary Cultured Prefrontal Cortex and Striatal Neurons

**Figure 2:**
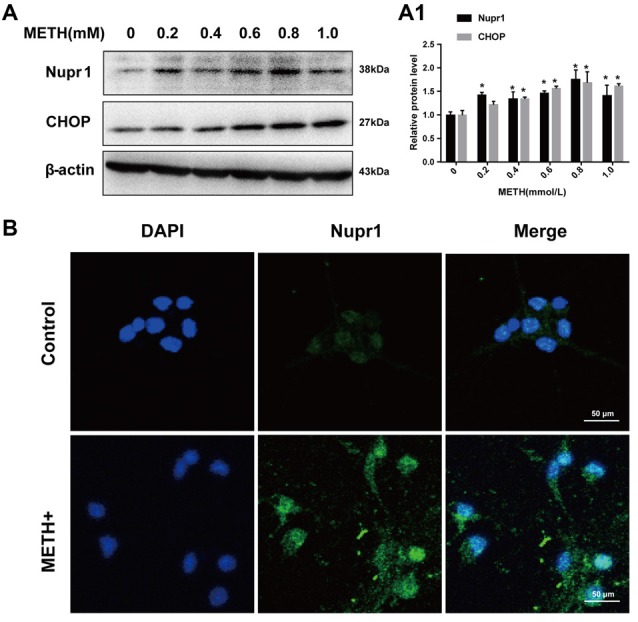
METH increases Nupr1 and CHOP protein expression in rat primary cultured prefrontal cortex and striatal neurons. METH exposure up-regulates Nupr1 and CHOP protein expression in a concentration- dependent manner in rat primary cultured prefrontal cortex and striatum neurons. Rat primary cultured prefrontal cortex and striatal neurons were exposed to 0.2 mM, 0.4 mM, 0.6 mM, 0.8 mM and 1.0 mM METH for 24 h, Western blot **(A)** and quantitative analyses **(A1)** were performed to determine Nupr1 and CHOP protein expression. Nupr1 was expressed at a higher level after 1 mM METH exposure than in the control group analyzed using a fluorescence microscope **(B)**. Nupr1 was stained with anti-p8 antibody (green); nuclei were counterstained with 4′,6′-diamidino-2-phenylindole (DAPI; blue). β-actin was used as a loading control. Fold induction relative to cells treated with vehicle is shown. *p < 0.05 vs. vehicle-treated cells. Data were analyzed with one-way ANOVA followed by LSD *post hoc* analyses. Data are expressed as means ± SD (*n* = 3/group).

Rat primary cultured prefrontal cortex and striatal neurons were exposed to 0.2 mM, 0.4 mM, 0.6 mM, 0.8 mM and 1.0 mM METH for 24 h. These concentrations were selected based on the LC25 (0.58 mM) of METH in rat primary culture neurons measured in our lab and also based on the concentrations used in other studies (Warren et al., 2007; Chou et al., 2008). These concentrations are similar to the concentrations used in PC12 cell experiments. Western blot showed that Nupr1 and CHOP protein levels were increased significantly (*n* = 3, *p < 0.05) by 30%–70% in a dose-dependent manner (Figures 2A,A1). Immunofluorescence staining results showed that Nupr1 expression was increased in primary cultured neurons after METH exposure (1.0 mM, 24 h; Figure 2B). These results suggest that METH exposure induces Nupr1 and CHOP protein expression in rat primary cultured prefrontal cortex and striatal neurons.

### METH Increases Nupr1 and CHOP Protein Expression *In Vivo*

**Figure 3:**
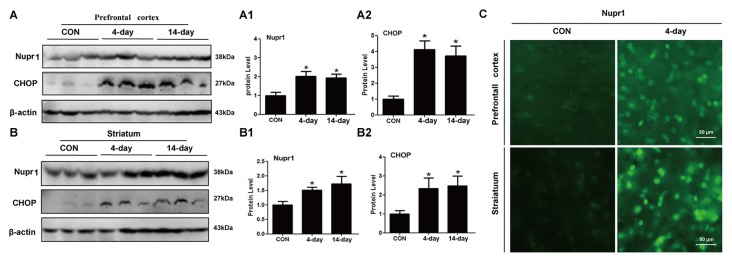
METH increases Nupr1 and CHOP protein expression *in vivo*. METH increased Nupr1 and CHOP expression in the prefrontal cortex and striatum of male Sprague-Dawley (SD) rats. Male SD rats were divided randomly into control group (saline vehicle), subacute group (15 mg/kg × 8 injections at 12 h intervals), and chronic exposure group (Table 1; *n* = 3/group). The prefrontal cortex **(A,A1,A2)**, striatum **(B,B1,B2)** tissues were harvested at 24 h after the last dosing. Western blot **(A,B)** and quantitative analyses **(A1,A2,B1,B2)** were performed to determine Nupr1 and CHOP protein expression. Immunofluorescence staining of rat brain sections showed Nupr1 expression was increased after METH exposure (15 mg/kg × 8 injections at 12 h intervals) **(C)**. Nupr1 was stained with anti-p8 antibody (green). β-actin was used as a loading control for Western blot analyses. Fold induction relative to the vehicle-treated group is shown. *p < 0.05 vs. the vehicle-treated group. Data in **(A1,A2,B1,B2)** were analyzed by one-way ANOVA followed by LSD *post hoc* analyses. Data are expressed as means ± SD (*n* = 3/group).

To test whether METH induces Nupr1 and CHOP expression *in vivo*, we determined Nupr1 and CHOP protein levels in the prefrontal cortex and striatum of rats following subacute or chronic METH exposure. Western blot results showed that in the prefrontal cortex Nupr1 protein level was 2.07-fold and 1.88-fold higher in the subacute and chronic exposure groups than in the control group, respectively (Figures 3A,A1,A2). This increase for CHOP protein level was 4.18-fold and 3.77-fold in the subacute and chronic exposure groups, respectively. Subacute and chronic METH exposures also increased Nupr1 (1.52 and 1.68-fold, respectively) and CHOP (2.41 and 2.55-fold, respectively) protein expression in the striatum (Figures 3B,B1,B2). Immunofluorescence staining results demonstrated that subacute METH treatment increased Nupr1 expression in both prefrontal cortex and striatum of rats (Figure 3C). These results suggest that METH exposure upregulates Nupr1 and CHOP protein expression, which may contribute to METH-induced ER stress *in vivo*.

### Silencing of Nupr1 Expression Attenuates METH-Induced Apoptosis and Autophagy in PC12 Cells

**Figure 4:**
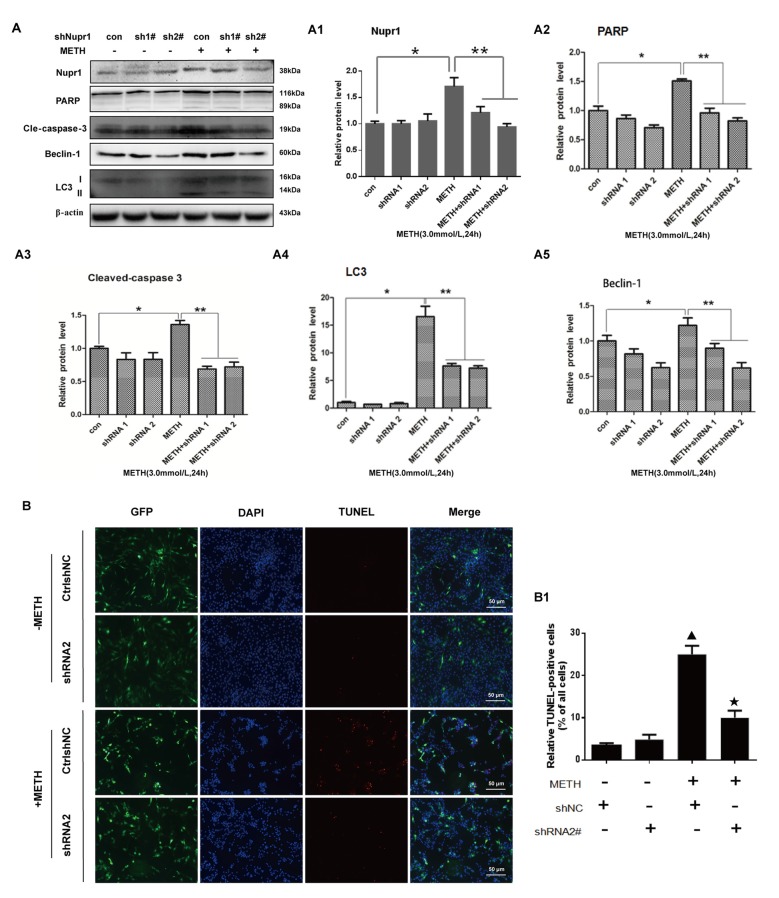
Silencing of Nupr1 expression reduces METH-induced apoptosis and autophagy in PC12 cells. PC12 cells were transfected with shNC or shNupr1 and then treated with or without METH (3.0 mM) for 24 h. Western blot **(A)** and quantitative analyses **(A1–A5)** were performed to evaluate the efficiency of Nupr1 knockdown, and the expression of apoptosis markers PARP and cleaved-caspase 3, and autophagy markers Beclin-1 and LC3 in PC12 cells. β-actin was used as a loading control. Fold induction relative to vehicle-treated cells is shown. *p < 0.05 vs. shNC- and vehicle-treated group. **p < 0.05 vs. shNupr1 and METH-treated group. Data in **(A1–A5)** were analyzed by one-way ANOVA followed by LSD *post hoc* analyses. Data are expressed as means ± SD (*n* = 3/group). **(B)** Cell apoptosis was evaluated with TUNEL staining. Apoptotic cells were stained with TUNEL (red). Nuclei were counterstained with DAPI (blue). **(B1)** Quantitative analysis of the percentage of apoptotic cells was performed with a standard cell counting method. The number of positive cells is presented as means ± SD (*n* = 3/group). ^▴^*p* < 0.05 vs. shNC- and vehicle-treated group. ^★^*p* < 0.05 vs. shNupr1 and METH-treated group.

To further assess the role of Nupr1 in METH-caused neurotoxicity, we investigated whether Nupr1 knockdown affects apoptosis and autophagy triggered by METH in PC12 cells using Western blot and TUNEL staining. We infected with lentivirus LV-shNupr1 #1 and LV-shNupr1 #2 or control lentivirus LV-GFP (100 nM) into PC12 cells for 48 h followed by METH (3.0 mM) exposure for 24 h. We observed that METH exposure significantly increased the expression levels of several proteins, including Nupr1, cell apoptosis markers PARP and cleaved caspase-3, as well as autophagy markers Beclin-1 and LC3 (Figures 4A,A1–A5). Notably, the expression levels of these proteins were all decreased to the control group level after co-exposure to METH and either one of the two shRNAs, with the effect of shNupr1 #2 being stronger. Therefore, shNupr1 #2 was chosen for subsequent TUNEL staining analysis. The results showed that the number of TUNEL-positive cells was increased by more than 8-fold in PC12 cells transfected with control shRNA and exposed to METH compared with vehicle treatment, and the number of TUNEL-positive cells was decreased by >2-fold in PC12 cells treated with METH and shNupr1 #2 compared with control shRNA group (Figures 4B,B1). These results suggest that silencing of Nupr1 expression can reduce apoptosis and autophagy induced by METH (3.0 mM) in PC12 cells.

### Silencing of Nupr1 Expression Decreases METH-Induced Increased Expression of CHOP and Trib3 in PC12 Cells

**Figure 5:**
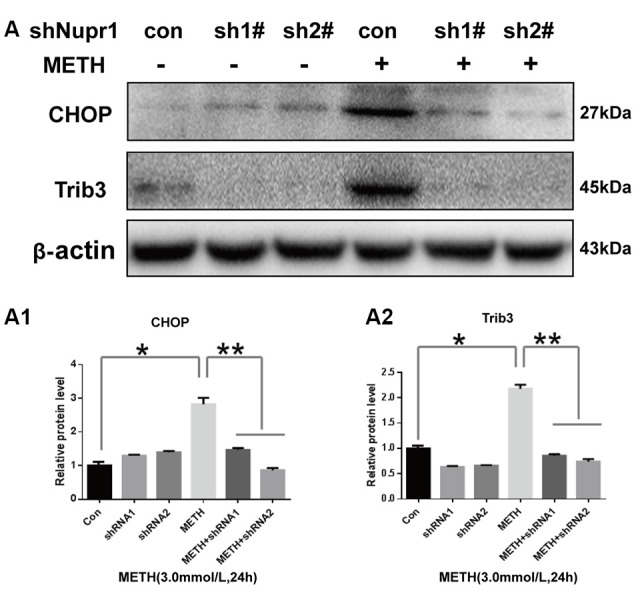
Silencing of Nupr1 expression decreases METH-induced CHOP and Trib3 expression in PC12 cells. PC12 cells were transfected with shNC or shNupr1 (shNupr1 #1 or shNupr1 #2) and then treated with or without 3.0 mM METH for 24 h. Western blot **(A)** and quantitative analyses **(A1,A2)** were performed to examine the activation level of CHOP and Trib3 expression in PC12 cells. Nupr1 knockdown decreases METH-induced expression of CHOP and Trib3 in PC12 cells. *p < 0.05 vs. shNC- and vehicle-treated group. **p < 0.05 vs. shNupr1 and METH-treated group. Data in **(A1,A2)** were analyzed by one-way ANOVA followed by LSD *post hoc* analyses. Data are expressed as means ± SD (*n* = 3/group).

Since METH exposure increased the expression of ER stress marker protein CHOP, indicating METH induces ER stress. Our next step was to determine whether Nupr1 is involved in METH-induced ER stress. We observed that METH exposure increased CHOP protein expression by 2.85-fold compared to the shNC group, and this increase was significantly attenuated to 50.9% and 30.1% by co-exposure of shNupr1 #1 and #2 sequences, respectively. Similar results were observed for Trib3 protein. METH exposure increased Trib3 protein expression by 2.21-fold compared to the shNC group, and this expression level was substantially decreased by 61.7% and 65.1% following co-exposure to shNupr1 #1 and #2 sequences, respectively (Figures 5A,A1,A2). These results suggest that Nupr1 may be an upstream regulator of CHOP and Trib3, which play a critical role in METH-induced ER stress.

### Silencing of CHOP or Trib3 Expression Reduces METH-Induced Apoptosis and Autophagy

**Figure 6:**
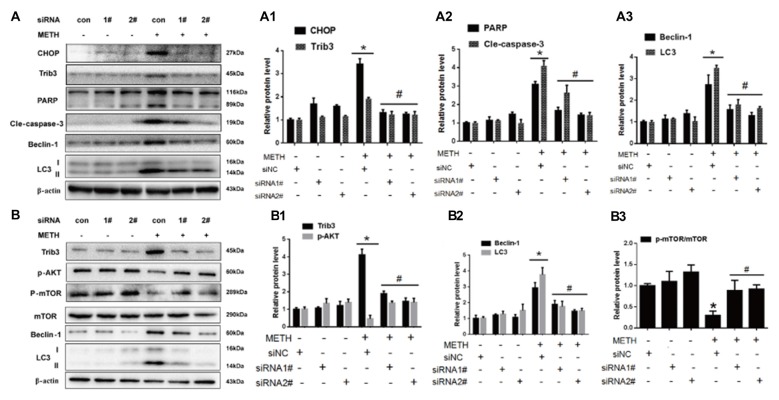
Silencing of CHOP or Trib3 reduces METH-induced apoptosis and autophagy in PC12 cells. PC12 cells were transfected with scrambled siRNA or corresponding CHOP and Trib3 siRNA for 48 h followed by treatment with or without METH (3.0 mM) for 24 h. Western Blot **(A,B)** and quantitative analyses **(A1–A3,B1–B3)** were performed to determine CHOP, Trib3, PARP, cleaved caspase-3, Beclin-1, LC3, p-AKT, p-mTOR and mTOR protein expression. β-actin was used as a loading control. Fold induction relative to vehicle-treated cells is presented. The experiment was performed in triplicate and the average fold change is shown. *p < 0.01 vs. siNC + vehicle group. ^#^*p* < 0.05 vs. siNC + METH group. Data are expressed as means ± SD (*n* = 3/group).

To confirm the role of CHOP and Trib3 in METH-induced apoptosis and autophagy, next we examined the activation level of apoptosis marker proteins PARP and cleaved caspase-3 and autophagy marker proteins Beclin-1 and LC3 after inhibition of CHOP or Trib3 expression by siRNA in PC12 cells followed by treatment with or without METH (3.0 mM). We transfected each of the two siRNA sequences (100 nM) or negative control siRNA (siNC; 100 nM), respectively, into PC12 cells for 48 h followed by METH (3.0 mM) exposure for 24 h. Western blot analyses showed that METH exposure increased CHOP protein expression by 3.55-fold compared to the siNC group, and this effect was significantly ameliorated to 40.3% and 35.7% by co-exposure to siCHOP #1 and #2 sequences, respectively. Similar results were observed for Trib3 proteins. METH exposure increased Trib3 protein expression by 1.98-fold; this effect was significantly mitigated by 40% after co-exposure to siCHOP #1 or #2 sequence (Figures 6A,A1). The results also showed that METH exposure significantly increased the expression of PARP, cleaved caspase-3, Beclin-1, and LC3 by 2.5-fold to 4-fold; this increase was diminished by 35%–65% after co-exposure to siCHOP #1 or #2 (Figures 6A,A2,A3). These results confirm that CHOP is involved in METH-induced neuronal apoptosis and autophagy and CHOP mediates METH-induced increase in the expression of Trib3.

Previous studies have shown that Trib3 is a novel ER stress marker protein that participates in Shiga toxins-caused autophagic cell death in intestinal epithelial cells (Tang et al., 2015). Trib3 is a downstream target of Nupr1. ER stress can upregulate the Nupr1/Trib3 pathway and then induce autophagy by inhibiting the AKT/mTORC1 axis (Salazar et al., 2009). In addition, phosphorylation of mTOR (p-mTOR) also plays an important role in cell autophagy (Meijer and Codogno, 2006; Chen R. et al., 2016). AKT, also known as protein kinase B, plays a critical role in controlling cell survival and apoptosis (Franke et al., 1997; Kandel and Hay, 1999). More importantly, AKT also plays a critical role in cell growth by directly phosphorylating mTOR (ser2448) in a rapamycin-sensitive complex containing raptor (Navé et al., 1999). Based on these facts, next we investigated whether Trib3 induces dopaminergic neuron autophagy via mTOR signaling pathway. We observed that METH exposure increased Trib3 protein expression by 4.17-fold compared to the siNC group, this effect was significantly attenuated by ~60% after co-exposure to siTrib3 #1 or #2 sequence. The phosphorylated AKT protein expression was decreased to 59.6% compared to the siNC group level, but its expression was elevated by ~3-fold after co-exposure to siTrib3 #1 or #2 (Figures 6B,B1). Consistent with the observation before, METH exposure increased the expression of autophagy marker proteins Beclin-1 and LC3-II by 3–4 folder. The expression level of Beclin-1 was reduced to 62.2% and 51.3% and the level of LC3-II was decreased to 47.7% and 41.3% after co-exposure to siTrib3 #1 and #2 sequences, respectively (Figures 6B,B2). Additionally, METH exposure decreased p-mTOR/mTOR protein expression ratio to 33.6% compared to the siNC group, this effect was normalized to the control group level after co-exposure to siTrib3 #1 or #2 (Figures 6B,B3). These results suggest that Trib3 is involved in METH-caused dopaminergic neuronal autophagy via inhibiting the mTOR signaling pathway.

### Silencing of Nupr1 Expression Reduces METH-Induced Apoptosis and Autophagy *In Vivo*

**Figure 7:**
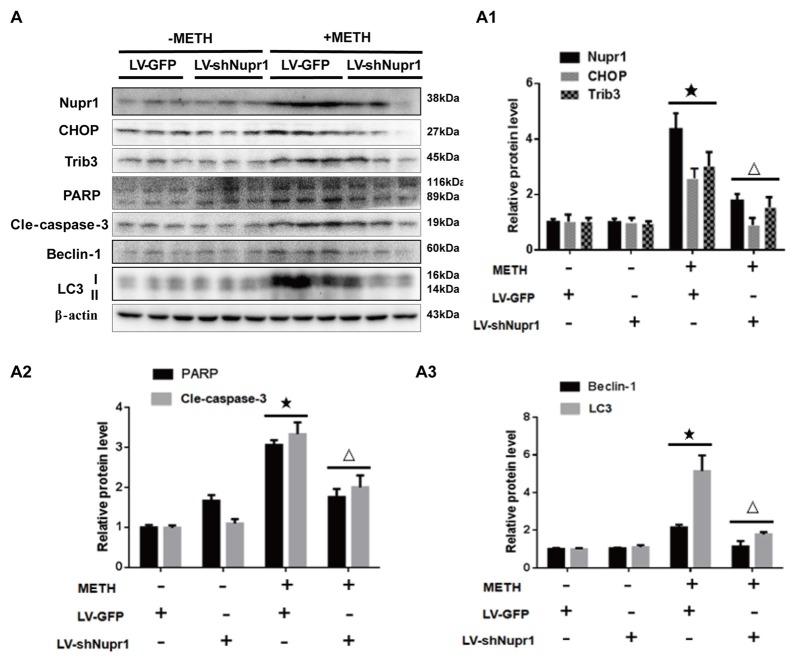
Silencing of Nupr1 expression reduces METH-induced apoptosis and autophagy *in vivo*. LV-GFP and LV-shNupr1 lentiviruses were injected separately to the right striatum of rats using a standard stereotaxic positioning system (*n* = 3/group). After 4 days of recovery, animals were injected intraperitoneally with saline or METH (15 mg/kg × 8 injections, at 12 h intervals). Striatal tissues (right side) were harvested at 24 h after the last dosing. Western blot **(A)** and quantitative analyses **(A1–A3)** were performed to determine Nupr1, CHOP, Trib3, cleaved caspase-3, PARP, Beclin-1 and LC3 protein expression. β-actin was used as a loading control. Fold induction relative to the vehicle-treated group is shown. ^★^*p* < 0.05 vs. LV-GFP and vehicle-treated group, ^△^*p* < 0.05 vs. LV-GFP- and METH-treated group. Data were analyzed by one-way ANOVA followed by LSD *post hoc* analyses. Data are expressed as means ± SD (*n* = 3/group).

To confirm the role of Nupr1 in METH-caused neurotoxicity *in vivo*, LV-GFP and LV-shNupr1 were injected separately to the striatum of rats using a standard stereotaxic positioning system to silence Nupr1 expression in the striatal region (*n* = 3/group). After stereotaxic injection and 4 days of recovery, rats were treated with saline or METH (15 mg/kg × 8 injections, at 12 h intervals). Western blot showed METH exposure induced Nupr1 protein expression to 4.81-fold higher than in the LV-GFP group; this effect was significantly attenuated to 38.5% by pre-injection with LV-shNupr1. Similar results were observed for CHOP and Trib3. Specifically, CHOP and Trib3 proteins were increased by 2.66-fold and 3.17-fold, respectively, after METH exposure, and their expression levels were decreased to 35.82% and 51.7%, respectively, when pre-treatment with LV-shNupr1 (Figures 7A,A1).

Next, we evaluated whether silencing Nupr1 expression can decrease METH-triggered apoptosis and autophagy in rats’ striatum. First, we measured PARP and cleaved caspase-3 protein levels in each treatment group. The results showed that cleaved PARP and cleaved caspase-3 protein levels were elevated by 3.11-fold and 3.43-fold, respectively, in METH-treated LV-GFP group, but were reduced to 59.8% and 58.7%, respectively, in the Nupr1 knockdown and METH treatment group (Figures 7A,A2). These results suggest that caspase-3 pathway is involved in Nupr1-mediated METH-induced dopaminergic neuronal apoptosis *in vivo*. We also measured the expression of autophagy marker proteins Beclin-1 and LC3 in the striatum of rats in different groups. After METH exposure, Beclin-1 and LC3-II protein levels were increased by 2.78-fold and 5.24-fold, respectively, but this effect was decreased by ~50%–60% in the METH + LV-shNupr1 group compared to the METH + LV-GFP group (Figures 7A,A3). All together, these results are consistent with those observed *in vitro* as described before and further demonstrated that Nupr1 plays an important role in METH-induced dopaminergic neuronal apoptosis and autophagy, and probably mediates METH-induced neurotoxicity through ER stress pathway.

## Discussion

In the present study, we report that Nupr1 expression is increased after a relatively high-dose METH exposure *in vivo* and *in vitro*. Nupr1 can promote dopaminergic neuronal apoptosis and autophagy through Nupr1/CHOP/Trib3 pathway induced by relatively high-dose METH, and silencing Nupr1 expression can partly prevent METH-induced apoptosis and autophagy *in vivo* and *in vitro*. These findings together with our previous study (Cai et al., 2016) indicate that Nupr1 plays a vital role in high-dose METH-induced toxicity in not only vascular endothelial cells, but also in dopaminergic cells.

Nupr1 as an stress gene functions in several biochemical pathways (Ree et al., 1999, 2000; Goruppi and Iovanna, 2010), and its induction by several signaling pathways indicates that Nupr1 may interact with different partner proteins and serve to potentiate different, or even opposing mechanisms depending upon the cellular context and stimulus (Mallo et al., 1997; Encinar et al., 2001; Goruppi and Iovanna, 2010). Recent studies have demonstrated that Nupr1 as a pro-survival gene while CHOP has pro-death functions associated with ER stress induced by calcitriol (Ozkaya et al., 2017). Nupr1 mediates its apoptotic effect through upregulation of the ER stress-related proteins ATF-4, CHOP and Trib3 (Carracedo et al., 2006). Moreover, sustained activation of Nupr1 and UPR-associated ER stress in ACOX1(−/−) mouse liver contributes to hepatocyte apoptosis (Huang et al., 2011). Our previous study indicate that Nupr1 plays as a pro-death protein participating in METH-induced apoptosis through Nupr1/CHOP pathway in vascular endothelial cells (Cai et al., 2016). Based on these findings, the present results confirm that Nupr1 is not only an stress-regulated protein, but also a pro-death protein participating in METH-induced apoptosis through the Nupr1/CHOP pathway in PC12 cells.

CHOP is a non-ER localized transcription factor that can be induced by a variety of adverse physiological conditions, including ER stress, and it is considered to be a critical mediator of stress-induced apoptosis (Kang, 2015). Upregulated expression of CHOP and induction of apoptosis in response to ER stress have been related to Nupr1 activation in astrocytoma cells exposed to cannabinoid (Carracedo et al., 2006). Our results show that knockdown the expression of Nupr1, METH-caused apoptosis is alleviated *in vitro* and *in vivo* and CHOP expression is also decreased following knockdown of the expression of Nupr1. Thus, Nupr1 serves as a pro-apoptosis protein upstream and regulates CHOP, promoting apoptosis caused by relatively high-dose METH exposure in PC12 cells. These findings suggest that Nupr1 plays a vital role in METH-induced apoptosis via Nupr1/CHOP pathway in PC12 cells.

Many studies have shown that METH can cause toxic effects to multiple organ systems (Darke et al., 2010; Carvalho et al., 2012; Vearrier et al., 2012; Halpin et al., 2014). Our previous studies (Cai et al., 2016; Chen R. et al., 2016; Wang et al., 2017) together with others (Fernandes et al., 2016; Bortell et al., 2017) have demonstrated that METH can cause apoptosis in endothelial cells, cardiomyocytes and hepatocytes, as well as induce astrocyte and microglial activation responses via different molecular signaling pathways in a dose-dependent manner that depends on the cell type. In this study, we mainly focused on the role of Nupr1 in neurotoxic effect of dopaminergic neurons after treatment with relatively high doses of METH. Although we have confirmed that Nupr1 acts via a similar mechanism in both vascular endothelial cells and dopaminergic PC12 cells, further studies are necessary to determine the role of Nupr1 in other types of cells.

The mechanism of ER stress underlying the neurotoxic effect of METH has been extensively studied and ER stress has been shown to activate multiple signaling pathways and mediate downstream pathophysiologic effects of METH exposure (Marciniak et al., 2004; Jayanthi et al., 2009; Takeichi et al., 2012). The presence of misfolded proteins in the ER triggers a cellular stress response called the UPR (Forman et al., 2003; Rutkowski and Kaufman, 2004; Hoozemans et al., 2005; Irie et al., 2011). The protein level of BiP/GRP78, a molecular chaperone which is up-regulated during the UPR, and can be used as a marker of ER stress (Forman et al., 2003; Moreno and Tiffany-Castiglioni, 2015; Casas, 2017; Shimizu et al., 2017). Based on our ongoing study (unpublished data), METH exposure can trigger ER stress and upregulate GRP78 protein expression in selected brain regions (e.g., striatum and prefrontal cortex) of METH-treated rats (15 mg/kg × 8 injections at 12 h intervals) compared to control animals. These results suggest that METH exposure causes ER stress in part by upregulating GRP78 protein expression.

Autophagy is an essential process for preserving cell homeostasis and basal autophagy seems to be present in almost all cell types; dysregulation of autophagy may contribute to several pathological conditions, leading to increased cell death (Klionsky and Emr, 2000; Mizushima and Komatsu, 2011; Eleftherios and Nicholas, 2016). METH is able to induce autophagy in dopaminergic neurons (Larsen et al., 2002; Castino et al., 2008). METH exposure induces intracellular inclusions in the nucleus and cytoplasm of striatal and substantia nigra neurons, and the same results have been observed in PC12 cells (Fornai et al., 2004). METH can also impair the autophagy–lysosome protein degradation system in HL-1 cultured mouse atrial cardiomyocytes (Funakoshi-Hirose et al., 2013). Up to now, there is no agreement on whether autophagy during METH-induced toxicity serves as pro-survival or pro-death function. A recent study shows that ER is essential for autophagosome formation (Hamasaki et al., 2013). Activation of ER stress can also affect autophagic flux, which may lead to cell death (Tripathi et al., 2016). Trib3, as a novel ER stress-inducible gene, is involved in autophagic cell death by inducing ER-stress, activating UPR reaction and through mTOR signaling pathway (Ohoka et al., 2005; Rubiolo et al., 2014). Studies have shown that upregulation of the expression of Trib3 can promote the inhibitory interaction of AKT with its upsteam kinases and lead to inhibition of AKT/mTORC1 axis and autophagy-mediated cell death (Salazar et al., 2015; Erazo et al., 2016). However, the role of Nupr1/Trib3 pathway underlying METH-induced autophagy in PC12 cells remains unclear.

In the present study, our results suggest that METH exposure increases the expression level of autophagy related marker protein Beclin-1 and LC3 in PC12 cells (3.0 mM), and also in the striatum of METH-treated rats, demonstrating that METH treatment can induce dopaminergic neuronal autophagy *in vivo* and *in vitro*. These outcomes are consistent with the findings from our previous and other studies (Kanthasamy et al., 2006; Kongsuphol et al., 2009; Nopparat et al., 2010; Li et al., 2017). We also demonstrated that relatively high-dose METH exposure increases the level of Nupr1 and Trib3, and knockdown of Nupr1 can partly downregulate the expression of Trib3 *in vivo* and *in vitro*. These findings suggest that Nupr1 as an upstream protein also regulates Trib3 expression in METH-induced autophagy in PC12 cells. Additionally, we found that METH-induced PC12 cell autophagy can alter AKT and mTOR activity by reducing p-AKT and p-mTOR expression in PC12 cells. This reduced expression of p-AKT and p-mTOR can be normalized after silencing Nupr1 expression. These results suggest that Nupr1 mediates dopaminergic neuronal autophagy triggered by METH partly via upregulating Nupr1 and Trib3 expression, followed by inhibiting p-AKT and p-mTOR activity. Taken together, these novel findings suggest that Nupr1 as a pro-autophagy protein plays a crucial role in relatively high-dose METH-induced autophagy in PC12 cells, and partly mediates autophagy through the Nupr1/Trib3/p-AKT/p-mTOR pathway.

The relationship between apoptosis and autophagy is very complex. Although in most cases, autophagy is thought to be a pro-survival mechanism, it also leads to cytotoxic effects and even cell death when the process exceeds a basal threshold (Mariño et al., 2014). Previous studies demonstrated that upregulated Beclin-1 level evoked by METH can form a complex with anti-apoptotic protein Bcl2, resulting in apoptosis (Nopparat et al., 2010; Ma et al., 2014). Activated caspase-3 can mediate the cleavage of Beclin-1 that facilitates the crosstalk between apoptosis and autophagy (Kang et al., 2011). In this study, we found that Beclin-1 and caspase-3 expression is increased after METH exposure *in vivo* and *in vitro*, and silencing Nupr1 expression can downregulate Beclin-1 and caspase-3 expression. However, the crosstalk mechanisms of how caspase-3 interacts with Beclin-1 in METH-induced apoptosis and autophagy need further research. Additionally, studies are also needed to confirm additional proteins involved in the interaction between Beclin-1 and caspase-3-mediated METH-induced neuronal cell apoptosis and autophagy. Our findings mainly provide the molecular mechanisms of Nupr1-mediated METH-induced apoptosis and autophagy *in vivo* and *in vitro*. Further research using Nupr1 knockout animal models are required to substantiate the present findings.

**Figure 8:**
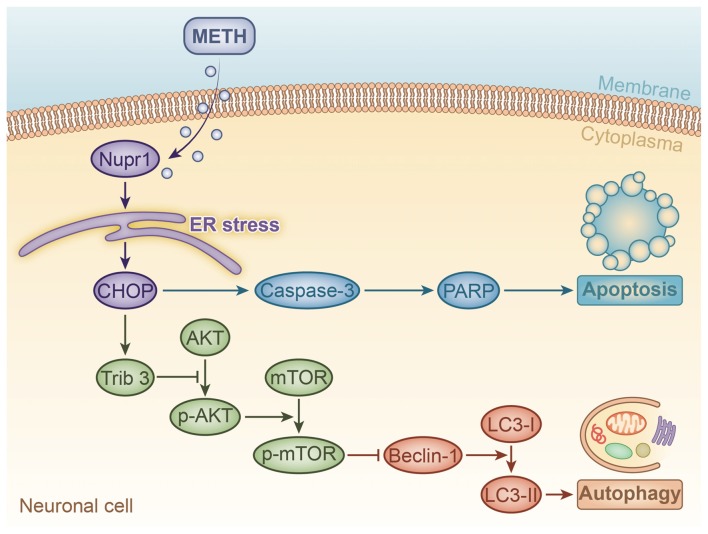
A schematic illustrating the role of Nupr1 in METH-induced apoptosis and autophagy in neuronal cells. METH exposure induces Nupr1 expression. Nupr1 mediates METH-induced apoptosis and autophagy in neuronal cells via triggering endoplasmic reticulum (ER) stress. CHOP as an ER stress marker protein can mediate METH-induced apoptosis through activating effector caspases, such as caspase-3, which is one of main executor targets to cleave PARP. Cleaved PARP facilitates cellular disassembly and serves as a marker of neuronal cell apoptosis. Trib3 is also an ER stress marker protein that can inhibit the phosphorylation of AKT and mTOR. As a classic inhibitor of autophagy, decreased phosphorylated mTOR increases the expression of Beclin-1, which activates the conversion of LC3-I to LC3-II, thereby inducing autophagy in neuronal cells.

In summary, our present study demonstrates that METH exposure at a relatively high-dose increases Nupr1 expression and activates ER stress-related proteins *in vitro* and *in vivo*. Nupr1 plays a pivotal role in dopaminergic neuronal apoptosis and autophagy induced by relatively high-dose METH. Nupr1 severs as a pro-apoptosis and pro-autophagy protein modulates METH-induced dopaminergic neuronal apoptosis and autophagy through CHOP-Trib3-mediated ER stress signaling pathway. Therefore, inhibition of Nupr1 expression may be a promising novel strategy to treat high-dose METH intoxication. Low-dose METH also induces dopaminergic neuronal apoptosis and autophagy, but does not cause significant change on Nupr1 expression. A schematic depicting this novel mechanism of the role of Nupr1 in METH-induced neuronal cell apoptosis and autophagy is provided in Figure 8. These findings provide insights into the molecular mechanisms of Nupr1-mediated METH-induced apoptosis and autophagy through ER stress signaling pathway in dopaminergic neuronal cells. Further studies are needed to determine the exact mechanisms of Nupr1-regulated neurodegenerative effects and the crosstalk in a dose- and time-dependent relationship between apoptosis and autophagy induced by METH.

## Author Contributions

XX and EH conducted all the experiments with the help of YT, XZ, XC, CC and RC. W-BX and HW designed the experiments. W-BX, HW, XX, CL and ZL analyzed and interpreted the results. W-BX and XX wrote the manuscript with the help of ZL.

## Conflict of Interest Statement

The authors declare that the research was conducted in the absence of any commercial or financial relationships that could be construed as a potential conflict of interest.
